# Presence of *Plasmodium falciparum* DNA in Plasma Does Not Predict Clinical Malaria in an HIV-1 Infected Population

**DOI:** 10.1371/journal.pone.0129519

**Published:** 2015-06-08

**Authors:** Marika Orlov, Laura M. Smeaton, Johnstone Kumwenda, Mina C. Hosseinipour, Thomas B. Campbell, Robert T. Schooley

**Affiliations:** 1 School of Medicine, University of California San Diego, San Diego, California, United States of America; 2 Center for Biostatistics in AIDS Research, Harvard School of Public Health, Boston, Massachusetts, United States of America; 3 Johns Hopkins University Research Project, Blantyre, Malawi; 4 Department of Medicine, University of North Carolina at Chapel Hill, Chapel Hill, North Carolina, United States of America; 5 University of North Carolina Project, Lilongwe, Malawi; 6 School of Medicine, University of Colorado, Aurora, Colorado, United States of America; University of California, San Francisco, UNITED STATES

## Abstract

**Background:**

HIV-1 and *Plasmodium falciparum* malaria cause substantial morbidity in Sub-Saharan Africa, especially as co-infecting pathogens. We examined the relationship between presence of *P*. *falciparum* DNA in plasma samples and clinical malaria as well as the impact of atazanavir, an HIV-1 protease inhibitor (PI), on *P*. *falciparum* PCR positivity.

**Methods:**

ACTG study A5175 compared two NNRTI-based regimens and one PI-based anti-retroviral (ARV) regimen in antiretroviral therapy naïve participants. We performed nested PCR on plasma samples for the *P*. *falciparum* 18s rRNA gene to detect the presence of malaria DNA in 215 of the 221 participants enrolled in Blantyre and Lilongwe, Malawi. We also studied the closest sample preceding the first malaria diagnosis from 102 persons with clinical malaria and randomly selected follow up samples from 88 persons without clinical malaria.

**Results:**

PCR positivity was observed in 18 (8%) baseline samples and was not significantly associated with age, sex, screening CD4+ T-cell count, baseline HIV-1 RNA level or co-trimoxazole use within the first 8 weeks. Neither baseline PCR positivity (p = 0.45) nor PCR positivity after initiation of antiretroviral therapy (p = 1.0) were significantly associated with subsequent clinical malaria. Randomization to the PI versus NNRTI ARV regimens was not significantly associated with either PCR positivity (p = 0.5) or clinical malaria (p = 0.609). Clinical malaria was associated with a history of tuberculosis (p = 0.006) and a lower BMI (p = 0.004).

**Conclusion:**

*P*. *falciparum* DNA was detected in 8% of participants at baseline, but was not significantly associated with subsequent development of clinical malaria. HIV PI therapy did not decrease the prevalence of PCR positivity or incidence of clinical disease.

## Introduction

HIV-1 and *Plasmodium falciparum* malaria are two of Sub-Saharan Africa’s major contributors to morbidity and mortality. Although the interaction between the two diseases was initially dismissed [1,2], more contemporary data support a bi-directionally deleterious interaction [3–7].

Although the majority of deaths from malaria are in children under 5 years of age, pregnant women and immunocompromised individuals (including those with HIV-1 infection) are also at increased risk for poor outcomes [8]. Adaptive immune responses developed during repetitive malaria episodes protect individuals from severe disease, however sterilizing immunity to *P*. *falciparum* infection never fully develops as individuals remain susceptible to both clinical disease and asymptomatic infections [9]. This asymptomatic carrier state has been termed subclinical (if the parasites are detectable in a blood smear) or subpatent (if the parasites are detectable by more sensitive methods such as PCR) parasitemia. While some studies suggest that subclinical and subpatent parasitemias are associated with the development of partial immunity and confer protection against future development of clinical disease [10,11], others suggest that the presence of asymptomatic parasitemia could itself be a marker for the development of clinical disease[12]. Very few such studies have been conducted in HIV infected populations [13,14].

HIV protease inhibitors (PIs) are in wide use for treatment of HIV infection in the US and Europe, but in resource limited settings, PIs are primarily reserved for second-line therapy. Although PIs inhibit replication of *P*. *falciparum* in tissue culture [15], their influence on the parasite *in vivo* has not yet been fully delineated. In one recently completed adult clinical trial comparing nevirapine-based (NNRTI) treatment to ritonavir-boosted lopinavir-based (PI) treatment, lopinavir therapy was not associated with lower rates of malarial antigen detection or clinical disease [16,17]. However a recently published trial in children showed a substantial decrease in clinical malaria incidence in the PI treated group [18]. In this study the reduction in malaria in the PI arm was observed only in the second and subsequent bouts of malaria and suggested that the overall reduction was driven primarily by increases in anti-malarial drug exposure caused by drug-drug interactions between PI’s and anti-malarial drugs used to treat the initial bouts of malaria in these children.

In the present study, we used a highly sensitive nested polymerase chain reaction (nPCR) to determine whether subpatent parasitemia was a predictor of clinical malaria and if treatment with atazanavir was associated with lower rates of subpatent parasitemia or clinical disease in study participants recruited to a clinical trial of antiretroviral therapy that was conducted in Malawi.

## Methods

IRB approval and informed consent were obtained from all participants in the parent study (see reference below). The UCSD Human Research Protections Program approved the use of the already collected patient samples in the ACTG study for this study.

### Patient population and sampling

PEARLS (Prospective Evaluation of Antiretrovirals in Resource limited settings, also known as ACTG A5175) randomized treatment-naïve individuals with CD4+ T-cells/mm^3^ counts of < 300 to lamivudine/zidovudine plus efavirenz, emtricitabine/tenofovir-DF plus efavirenz, or didanosine plus emtricitabine plus atazanavir (not ritonavir boosted) [19]. PEARLS recruited 1571 participants in 9 countries and included sites in Blantyre and Lilongwe, Malawi. During a median follow up period of three years, all but one of the 245 bouts of clinical malaria in the trial occurred in the 221 study participants recruited by the two sites in Malawi. We thus concentrated our evaluation of subpatent *P*. *falciparum* parasitemia on Malawian study enrollees. Cases of malaria were prospectively defined as ‘confirmed’ when study participants presented for clinical care with a compatible clinical syndrome (as determined by the individual investigator at each site) and were found to have *Plasmodium* sp. parasites on a peripheral blood smear and as ‘probable’ when study participants presented with a compatible clinical syndrome and specific treatment for *P*. *falciparum* was initiated or recommended without laboratory confirmation by a positive blood smear.

**Fig 1:**
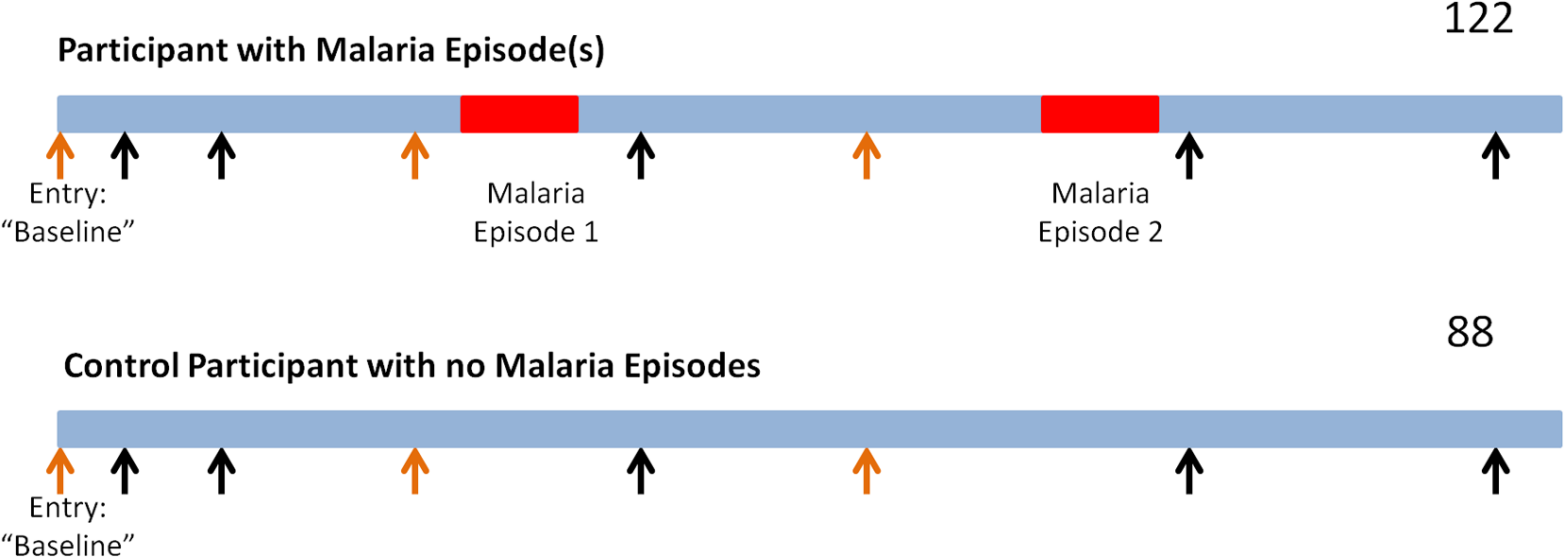
Schematic Diagram of Sampling Strategy. Of all the plasma samples that were collected for the PEARL study (all arrows), we used a subset of those samples for our study (orange arrows). We assayed all available baseline samples which were collected at entry into the antiretroviral study (215), the closest sample banked prior to a malaria episode (102), and control plasma samples from participants that did not develop malaria matched for time of year of sampling and length of time on antiretroviral therapy (88).

Plasma samples were obtained prior to study entry and at regular intervals during follow-up based on the sampling schedule dictated by the clinical trial. The sampling strategy for the evaluation of *P*. *falciparum* parasitemia is illustrated schematically in Fig 1. Of the available plasma samples collected during the antiretroviral chemotherapy study (all arrows), we selected a subset for analysis for the presence of *P*. *falciparum* DNA (subpatent parasitemia, orange arrows). Baseline samples were defined as those obtained prior to the initiation of antiretroviral therapy (N = 113) or within 8 weeks following entry into the clinical trial (N = 102). Two participants were removed from the analysis because they had active malaria at entry into the ARV study. Among the 18 study participants with positive baseline samples, another sample obtained 4–12 weeks later was requested to test for persistence of subpatent parasitemia. For the 122 participants who had a clinical malaria diagnosis during the study, PCR testing for subpatent parasitemia was also performed on the closest available specimen prior to the first diagnosis of clinical malaria. These samples were drawn an average of 31 days (range 1–60 days) prior to the malaria diagnosis. For those who never had a clinical malaria diagnosis (controls, 88), a 2-stage restricted sampling design was used to control for 2 potential confounding factors: time of year (high malaria season [November through May] versus low [remainder of year]) and exposure time to ARVs. Time since ARV initiation was divided into four intervals: <12 weeks, 12–24 weeks, 24 - < 96 weeks, and 96+ weeks. “Early” co-trimoxazole use refers to administration in the first 8 weeks of study.

### DNA extraction and PCR

200μL of plasma were used to extract DNA using Qiagen Mini Blood Kit (Qiagen) as per manufacturer’s instructions. The nested PCR (nPCR) for *P*. *falciparum* detects a 205bp portion of the 18s rRNA gene. A nested PCR reaction was performed on 40μL of supernatant from the extraction in a 50μL PCR reaction (2.5 U Taq polymerase (Invitrogen), 3mM MgCl_2_, 500nM dNTPs (Fermentas), and 50 pmol of each primer (IDT). In the first round of the nPCR, the gene was amplified using primers fPLU: 5’-TTA AAA TTG TTG CAG TTA AAA CG-3’, and rPLU: 5’-CCT GTT GTT GCC TTA AAC TTC-3’. Denaturation was performed at 94°C for 3 minutes, followed by 30 cycles of denaturation at 94°C for 30 seconds, annealing at 58°C for 1 minute, and elongation at 72°C for 2 minutes, with a final extension at 72°C for 5 minutes. 2μL of the outer PCR product was used as the template for the inner PCR reaction (50μL total) using primers fFAL: 5’-TTAAACTGGTTTGGGAAAACCAAATATATT-3’ and rFAL: 5’-ACACAATGAACTCAATCATGACTACCCGTC-3’. For the inner PCR, denaturation was performed at 94°C for 3 minutes, followed by 35 cycles of denaturation at 94°C for 30 seconds, annealing at 58°C for 1 minute, and elongation at 72°C for 1 minute, with a final extension at 72°C for 5 minutes. The PCR products from the inner PCR were run on a 1% agarose gel (175V, 30 minutes) and stained with ethidium bromide. The PCR result was qualitative and could detect parasite DNA in the plasma down to 1.8 parasite genomes/5μL of extracted DNA. The sensitivity threshold was defined by performing serial dilutions starting from a known parasitemia, followed by DNA extraction and then PCR.

### Statistical Analysis

Categorical variables were compared by Fisher’s exact test and continuous variables were compared with Wilcoxon Rank-Sum Test. Univariable logistic regression examined the association between subpatent parasitemia at baseline (or during follow-up) and clinical malaria diagnosis during study follow-up. To control for confounders, effect modifiers, and other prognostic factors, multi-variable logistic regression was used including covariates identified as associated with either development of clinical malaria during follow-up, or with subpatent status. Adding subpatent status tested for independent association provided by a plasma DNA finding at baseline. Since all of the participants placed on atazanavir were switched to NNRTI therapy after a median of 102 weeks in the trial and some participants were crossed over from NNRTIs to PIs or vice versa due to HIV outcome failures over the course of the study, the effect of PI therapy on subpatent parasitemia and clinical malaria outcomes were investigated using an as-treated analysis. Because we did not see any effect of drug therapy on subpatent parasitemia or clinical malaria outcomes, an intent-to-treat analysis was used for the remainder of our analysis.

## Results

### Subpatent Parasitemia

Of the 219 participants analyzed, 147 (67%) were female and 72 (33%) were male and the mean age was 33 (range 18–65). PCR positivity was observed in 18 (8.4%, 95% CI 5.0 to 12.9%) of 215 baseline samples. There was a trend toward a greater likelihood of PCR positivity in those with a lower BMI (p = 0.114) and lower hemoglobin levels (p = 0.066). There was no significant association between PCR positivity and early cotrimoxazole use, age, gender, CD4+ T-cell count or plasma HIV-1 RNA levels. None of the 37 participants with a prior tuberculosis diagnosis were found to have PCR positivity (p = 0.027) (Table 1).

**Table 1:** Correlates of Baseline Subpatent Malaria.

		No Subpatent Parasitemia(N = 197)	Subpatent Parasitemia(N = 18)	OR* (95% CI; p-value)
Malaria Season of Sample	High (Rainy)	93 (86%)	12 (14%)	1.90 (0.72–5.03; p = 0.189)
	Low (Dry)	104 (95%)	6 (5%)	
BMI	Mean (s.d.)	22 (4)	21 (3)	**0.88 (0.74–1.04; p = 0.114)**
Hemoglobin Value(g/dL)	Mean (s.d.)	12 (2)	11.2 (1.7)	**0.83(0.65–1.05; p = 0.066)**
Any History of TB	No	160 (90%)	18 (10%)	**0.11(0.01–1.92; p = 0.027)**
	Yes	37 (100%)	0 (0%)	
CD4+ Cell Count	Mean (s.d.)	169 (79)	184 (66)	1.00(1–1.01; p = 0.298)
Plasma HIV-1 viral load(log10 copies/mL)	Mean (s.d.)	5.0 (1)	5.0 (1)	0.76(0.40–1.43; p = 0.406)
Age	Mean (s.d.)	33 (8)	33 (8)	1.01(0.95–1.07; p = 0.721)
Gender	Male	64 (90%)	7 (10%)	0.65(0.25–1.70; p = 0.386)
	Female	133 (92%)	11 (8%)	
Early TMP/SMX use	Yes	24 (96%)	1 (4%)	1.20(0.45, 3.20; p = 0.713)
	No	173 (91%)	17 (9%)	
Randomization to NNRTI arm	NNRTI	130 (92%)	11 (8%)	0.70(0.27–1.82; p = 0.466)
	PI	67 (91%)	7 (9%)	

*OR values are not adjusted.

Of the 219 participants, 56% (122) developed probable (28/122) or confirmed (94/122) clinical malaria. Neither PCR positivity at baseline (p = 0.45) nor during follow-up visits (p = 1.0) was associated with the subsequent development of confirmed or probable malaria over the course of the study. 50% of those with PCR positivity at baseline had a bout of clinical malaria within the first 24 weeks while 24% of those without PCR positivity at baseline had clinical malaria in the same time period (positive predictive value of 50%, 95% CI: 26–74). The sensitivity of PCR positivity for predicting clinical malaria in this interval was only 16% (95% CI: 8–23).

**Fig 2:**
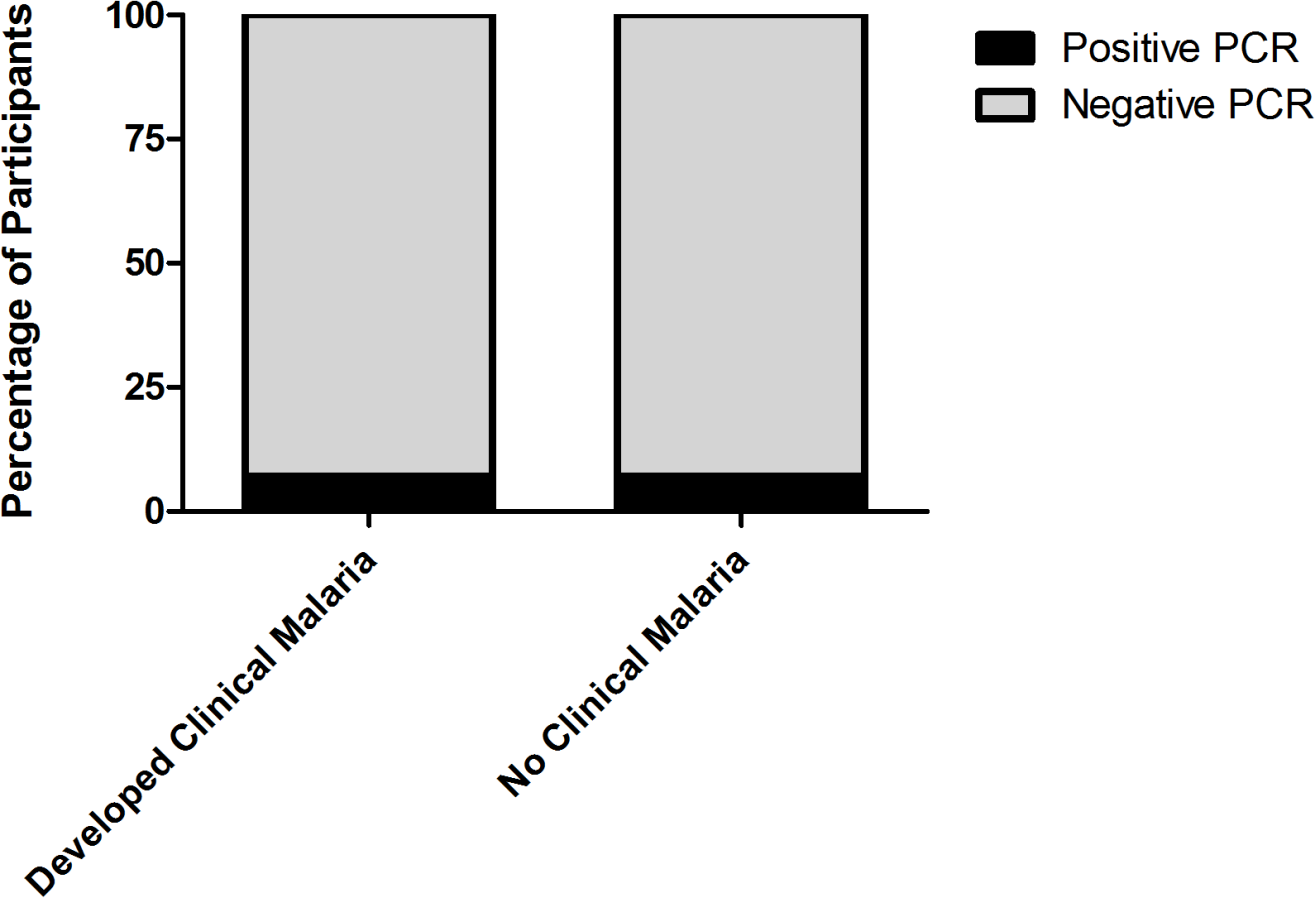
Detection of Subpatent Parasitemia does not predict development of clinical disease. Subpatent parasitemia is not a good predictor of development of clinical disease. Among the 102 cases of malaria for whom plasma was available for PCR preceding diagnosis, subpatent parasitemia was detected in only 7 (6.9%). Among 88 controls without subsequent clinical malaria, 6 (6.8%) had subpatent parasitemia detected. Levels of subpatent parasitemia were equivalent in the participants that went on to develop clinical disease to those that never developed clinical malaria over the course of the study.

**Fig 3:**
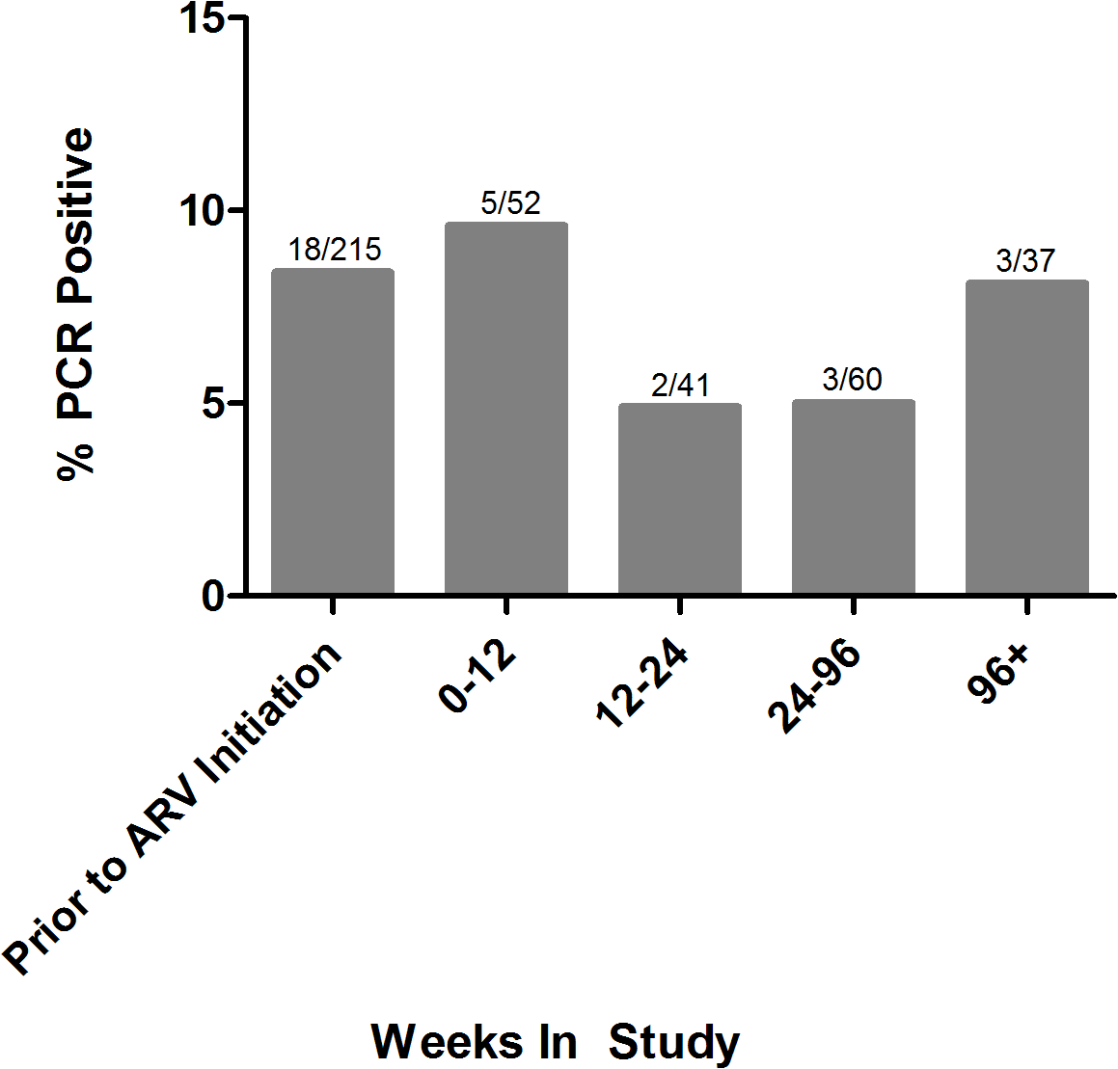
Prevalence of Subpatent Parasitemia Over the Course of the Study. We analyzed 215 baseline plasma sample, the closest available plasma sample prior to the first malaria diagnosis for 102 participants, and a stratified random sample from 88 study participants who never had a malaria diagnosis. PCR positivity remained relatively stable (5–10%) over the course of the study in the samples that we assayed.

PCR positivity was detected in only 7 (6.9%) of the closest plasma samples collected prior to the onset of the first bout of clinical malaria in 102 individuals during the trial. Among the 88 controls that were selected because they did not experience clinical malaria during the study, PCR positivity was detected at the same frequency (6.8%) (Fig 2), further suggesting that subpatent parasitemia was not a useful predictor of clinical disease (p = 1.0) in this patient population. Importantly, PCR positivity was not associated with length of time that had elapsed between plasma collection and clinical diagnosis among those with a clinical malaria episode (p = 0.6). Randomization to the atazanavir arm was not significantly associated with a decrease in subpatent parasitemia compared to those assigned to the efavirenz arms (OR 1.23, 95% CI 0.46–3.33, p = 0.7). In samples that were tested after the initiation of ARV therapy, the prevalence of PCR positivity was relatively stable over the 3 year study (5–10%) (Fig 3).

PCR positivity persisted after the initiation of antiretroviral therapy. If a baseline sample was PCR positive, we continued testing follow up samples from the same individual until they were no longer positive. Of the 18 positive samples at baseline, a follow-up sample was available for 17; 76% (13/17) of these samples remained positive (follow-up samples obtained 4–8 weeks after baseline). Among the 13 remaining positive at the second determination, only 30% (4/13) remained positive when a third sample collected 1–8 weeks later was tested. The patterns of persistent PCR positivity were not associated with NNRTI use (p = 1.0) or early co-trimoxazole use (p = 1.0). Persistent positivity was not associated with a diagnosis of clinical malaria over the course of the trial (confirmed p = 0.3; probable and confirmed p = 0.8).

### Clinical Malaria

A total of 122 of the 219 participants (56%) had at least one probable or confirmed malaria diagnosis during the course of the three-year study, for a total of 238 clinical events. Clinical malaria (confirmed or probable) was observed more frequently in those with history of tuberculosis (OR 2.91, 95% CI 1.30–6.52, p = 0.006) and with a lower body mass index (OR 0.89, 95% CI 0.82–0.97, p = 0.004). Those using cotrimoxazole in the first 8 weeks were more likely to develop clinical malaria (OR 1.80, 95% CI 1.01–3.23, p = 0.045) but this association did not persist once adjusted for baseline BMI and TB history. Other baseline characteristics including CD4+ T-cell count, plasma HIV-1 RNA level and hemoglobin levels were not significantly associated with development of clinical malaria during the study (Table 2). Confirmed clinical malaria was only associated with baseline BMI (OR 0.89, 95% CI 0.82–0.97, p = 0.007). Finally, ARV regimen was not significantly associated with confirmed or probable clinical malaria (OR = 1.16, 95% CI 0.66–2.04).

**Table 2:** Correlates of Probable and Confirmed Clinical Malaria.

		None (N = 97)	Probable(N = 28)	Confirmed(N = 94)	OR*(95% CI; p-value)
BMI	Mean (s.d.)	23 (4)	22 (3)	21 (3)	**0.89(0.82–0.97; p = 0.004)**
Screening CD4+ Cell Count	Mean (s.d.)	168 (78)	169 (83)	173 (78)	1.00(1.0–1.0; p = 0.702)
Plasma HIV-1 viral load (log10 copies/mL)	Mean (s.d.)	5 (1)	5 (1)	5 (1)	1.13(0.77–1.67; p = 0.532)
Early TMP/SMX use	No	72 (49%)	17 (12%)	58 (39%)	**1.80(1.01–3.23; p = 0.045)**
	Yes	25 (35%)	11 (15%)	36 (50%)	
Any History of TB	No	88 (48%)	22 (12%)	72 (40%)	**2.91(1.30–6.52; p = 0.006)**
	Yes	9 (24%)	6 (16%)	22 (60%)	
Hemoglobin Value(g/dL)	Mean (s.d.)	12 (2.1)	11.8 (1.8)	11.9 (1.9)	0.97(0.84–1.11; p = 0.658)
Randomized Treatment Group	NNRTI	66 (46%)	22 (15%)	57 (39%)	1.16(0.66–2.04; p = 0.609)
	PI	31 (42%)	6 (8%)	37 (50%)	

*OR values are not adjusted.

## Discussion

Over the past decade, there has been substantial discussion and debate about the extent to which *P*. *falciparum* co-infection influences the morbidity and mortality of HIV disease and *vice versa*. As additional data have accumulated it appears that some of the contradictory findings fueling this debate have been driven by the dynamic and complex interplay among these two pathogens and the human immune system and are heavily influenced by the underlying prevalence of each of the two pathogens in a given population [20,21]. We studied a population of adults in a region hyperendemic for malaria immediately before and after initiating antiretroviral therapy to better understand this interplay. At the time when the PEARLS trial was initiated in 2005, the districts of Blantyre and Lilongwe together had 23% of the total new malaria cases in Malawi, with the number of malaria cases being 27% and 21% of the total percent of the population, respectively [22]. The incidence of malaria cases was found to be higher in females and in individuals with HIV [22]. Given that our sub-study population was 67% female and all of the participants had CD4+ T-cell counts less than 300/mm^3^, it is not surprising that about half of the participants had documented clinical events and some of them had more than 1 event over the course of the trial. Our primary goal was to determine whether the detection of *P*. *falciparum* DNA in plasma samples obtained from those with relatively advanced HIV-associated immunodeficiency was a predictor of clinical malaria or, alternatively, that PCR positivity in the absence of clinical disease identifies partially immune individuals who have the capacity to immunologically control the organism.

In our study we found that PCR positivity was not significantly associated with subsequent development of clinical malaria. Neither lower pre-entry CD4+ T-cell counts nor higher plasma HIV-1 RNA levels at study entry were significantly associated with either PCR positivity or clinical malaria. While other studies have documented an association between lower CD4+ T-cell counts and increased episodes of malaria, these studies included patients with a relatively wide range of CD4+ T-cell counts and ours was restricted to candidates for antiretroviral therapy and composed only of those with CD4+ T-cell counts below 300/mm^3^[23]. We did find that presence of *P*. *falciparum* DNA in the plasma at study entry and development of clinical malaria were more common in those with a lower BMI. PCR positivity at study entry was also more common in those with lower hemoglobin levels and in those with no prior history of TB. In contrast, development of probable or confirmed clinical malaria was positively associated with a history of TB. Although these seemingly contradictory findings are driven by relatively small numbers of patients, one could speculate that those without a prior history of tuberculosis were more immunologically competent and, thus, able to maintain *P*. *falciparum* in a subpatent state while those with a prior history of tuberculosis were more likely to develop clinical disease when they were exposed to the parasite.

In addition, we did not observe an association between HIV protease inhibitor therapy and PCR positivity or clinical malaria in our small study. Andrews, *et al*, demonstrated that several PIs, including atazanavir, reach concentrations in the plasma that exceed the EC50 for anti-malaria activity *in vitro* [24]. Sera from subjects taking PIs have been reported to have antimalarial activity *in vitro*, supporting the possibility that PIs reach levels in the range required for anti-malarial activity [25,26]. Atazanavir blood levels in the PEARLS study (28nM–14μM) [27] overlapped those required to inhibit parasite growth *in vitro* (2.5–11.6 μM) [15], though atazanavir blood levels in some participants in the trial would have been lower than those required to suppress malarial parasites *in vitro*. In a recently reported study of 745 HIV-infected African women, ritonavir boosted lopinavir did not decrease the incidence of clinical malaria or subpatent parasitemia (detected using a malaria rapid antigen test) [16,17]. In another study in children, although PIs were not associated with a lower incidence of initial bouts of malaria, subsequent bouts were delayed [18]. This reduction in clinical disease among those on HIV protease inhibitors was attributed by the investigators to increased persistence of antimalarial drugs following treatment of the initial bout of malaria which was driven by drug-drug interactions between the protease inhibitors and the antimalarial drugs used in the study.

In conclusion, detection of *P*. *falciparum* DNA in this population of HIV-1 infected adults residing in a hyperendemic area for malaria was not significantly associated with subsequent clinical disease. Our study has a number of important limitations. First, the study was retrospective using available samples from a previously completed antiretroviral drug study. Although many cases of malaria were reported among the Malawi participants, plasma samples were not routinely collected at the time of the malaria episode. The samples available were collected at study entry and at regular intervals during the study for the purpose of assessing the efficacy of antiretroviral therapy. Although we did not see an association between parasitemia and the amount of time elapsed to subsequent diagnosis of clinical malaria, the samples were collected, on average, 31 days prior to the malaria diagnosis. The low prevalence of baseline and follow-up PCR positivity and clinical malaria cases limited the statistical power to detect the effect of the PI-based therapy. Lastly, we had access to frozen plasma samples, not blood spots. Although others have previously reported that parasite DNA can be found in the plasma of patients with clinical malaria [28,29], the use of this approach to evaluate the prevalence of subclinical or subpatent *P*. *falciparum* infection has not been previously reported. The quantitative and qualitative relationships between levels of detection of *P*. *falciparum* DNA in blood (or dried blood spots) compared to plasma have not been extensively studied. However, in one study using quantitative PCR, parasite DNA was detected in blood in 3.5-fold more samples than in plasma [30]. Although the comparison was complicated by the use of different primer/probe sets for the detection of parasite DNA in the blood versus the plasma, it is biologically probable that blood samples would be more sensitive than plasma samples for the detection of plasmodium DNA. A similar comparison of the sensitivity of nested PCR has not yet been reported. Nonetheless, the use of frozen plasma samples for studies of malarial prevalence could open doors to the study of changes in prevalence of the parasite over time in archived samples collected for other reasons–especially in the context of malaria eradication studies. Most of the prior studies that have addressed the impact of *P*. *falciparum* infection on HIV-1 and vice versa have been conducted using clinical evidence of malaria as a marker for *P*. *falciparum*. Larger scale longitudinal studies of PCR positivity and of clinical disease in regions of varying malaria endemnicity, in additional HIV-infected populations, are needed to more conclusively examine the clinical implications of PCR positivity and of the impact of PCR positivity on the natural history of HIV-1 infection. Finally such studies, when combined with more focused studies of malaria-specific immunity, could provide important insights into the immunological correlates of protective immunity.
